# Prophage-Dependent Neighbor Predation Fosters Horizontal Gene Transfer by Natural Transformation

**DOI:** 10.1128/mSphere.00975-20

**Published:** 2020-11-11

**Authors:** Roberto C. Molina-Quiroz, Triana N. Dalia, Andrew Camilli, Ankur B. Dalia, Cecilia A. Silva-Valenzuela

**Affiliations:** a Centro de Estudios Científicos, Valdivia, Los Rios, Chile; b Department of Biology, Indiana University, Bloomington, Indiana, USA; c Department of Molecular Biology and Microbiology, Tufts University, School of Medicine, Boston, Massachusetts, USA; University of Michigan-Ann Arbor

**Keywords:** HGT, *Vibrio cholerae*, bacteriophages, chitin, natural transformation, neighbor predation

## Abstract

Natural transformation is a broadly conserved mechanism of horizontal gene transfer (HGT) in bacteria that can shape their evolution through the acquisition of genes that promote virulence, antibiotic resistance, and other traits. Recent work has established that neighbor predation via type VI secretion systems, bacteriocins, and virulent phages plays an important role in promoting HGT. Here, we demonstrate that in chitin estuary microcosms, Vibrio cholerae K139 lysogens exhibit prophage-dependent neighbor predation of nonlysogens to enhance HGT. Through predation of nonlysogens, K139 lysogens also have a fitness advantage under these microcosm conditions. The ecological strategy revealed by our work provides a better understanding of the evolutionary mechanisms used by bacteria to adapt in their natural setting and contributes to our understanding of the selective pressures that may drive prophage maintenance in bacterial genomes.

**IMPORTANCE** Prophages are nearly ubiquitous in bacterial species. These integrated phage elements have previously been implicated in horizontal gene transfer (HGT) largely through their ability to carry out transduction (generalized or specialized). Here, we show that prophage-encoded viral particles promote neighbor predation leading to enhanced HGT by natural transformation in the waterborne pathogen Vibrio cholerae. Our findings contribute to a comprehensive understanding of the dynamic forces involved in prophage maintenance which ultimately drive the evolution of naturally competent bacteria in their natural environment.

## Abstract (precis)

Prophages are nearly ubiquitous in bacterial species. These integrated phage elements have previously been implicated in horizontal gene transfer (HGT) largely through their ability to carry out transduction (generalized or specialized). Here, we show that prophage-encoded viral particles promote neighbor predation leading to enhanced HGT by natural transformation in the waterborne pathogen Vibrio cholerae. Our findings contribute to a comprehensive understanding of the dynamic forces involved in prophage maintenance which ultimately drive the evolution of naturally competent bacteria in their natural environment.

## OBSERVATION

Several bacterial species have evolved to capture DNA (natural competence) as a source of nutrients (1) or to incorporate it into their genome to speed their evolution via a process termed natural transformation (NT) (14–16). Vibrio cholerae is a genetically tractable and well-established model organism to study NT. This human pathogen is usually found in association with the chitinous carapaces of zooplankton in estuary and ocean waters (2). V. cholerae can utilize chitin as a major carbon and nitrogen source, and additionally, this polymer is a required signal for induction of NT (3). In estuarine chitin microcosms, it has been shown that this pathogen can take up multiple large DNA fragments when the exogenous DNA concentration is high (4, 5). Much evidence points to NT and horizontal gene transfer (HGT) having contributed to the evolution of V. cholerae (6).

About 50% of V. cholerae clinical isolates carry the temperate kappa phage K139 (7). However, the role of K139 in the ecology of V. cholerae has been ill defined. Here, we explore whether the lytic replication of K139 affects the physiology of V. cholerae in chitin microcosms, which mimic the aquatic reservoir for this facultative pathogen.

**FIG 1:**
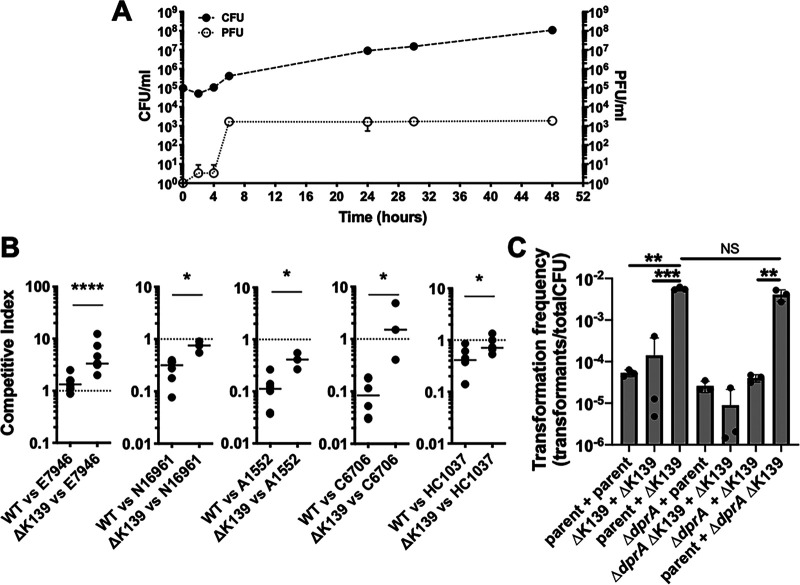
K139 promotes neighbor predation and enhances HGT by NT in chitin microcosms. (A) V. cholerae E7946 growth (CFU, solid circles) and K139 phage titer in culture supernatants (PFU, empty circles) were measured in chitin microcosms for 48 h. (B) Competition between V. cholerae strains mixed 1:1 was assessed following 24 h of growth in chitin microcosms. All competitions were performed between the indicated clinical isolate (E7946, N16961, A1552, C6706, or HC1037) and an E7946 lysogen (WT) or an E7946 nonlysogen (ΔK139). The competitive index is reported as the ratio of clinical isolate to E7946 WT or clinical isolate to E7946 ΔK139 in the output divided by the same ratio in the input. Data are from 4 independent experiments, and the line within samples denotes the median. The dotted line indicates a CI of 1, which is the value expected if strains compete equally. Statistical comparisons were made by Mann-Whitney test (*, *P* < 0.05; ****, *P* < 0.0001). (C) The indicated variants of V. cholerae E7946 were cocultured in chitin microcosms at 30°C for 48 h to assess HGT, which is reported as the transformation frequency (see text for details). Data represent three independent biological replicates shown as the mean ± SD. Statistical comparisons were made by one-way analysis of variance (ANOVA) with Tukey’s posttest on the log-transformed data (**, *P* < 0.01; ***, *P* < 0.001; NS, not significant).

Using the K139 lysogen E7946, an O1 El Tor V. cholerae strain, we first evaluated bacterial replication and production of viral particles over time in chitin microcosms. V. cholerae growth was slow, and bacterial numbers increased 100-fold by 24 h and 1,000-fold by 48 h of incubation, while K139 PFU increased 3 logs by 6 h, which is when bacteria were entering exponential growth (Fig. 1A). Interestingly, we found that insoluble chitin specifically increased K139 PFU compared to other carbon sources (see Fig. S1 in the supplemental material). Furthermore, addition of alternative carbon sources (e.g., glucose) to chitin microcosms reduced K139 PFU (Fig. S1), suggesting that phage production may be regulated by carbon catabolite repression. Based on these results, we hypothesized that K139 might have a role in the ecology of V. cholerae in its environmental reservoir by increasing the competitive fitness of lysogenic strains. To evaluate if K139 is able to kill nonlysogenic strains in cocultures, E7946 (lysogen, wild type [WT]) and an isogenic E7946 ΔK139 mutant (nonlysogen) were competed against a lysogen (E7946 itself) and a panel of diverse clinical isolates that naturally lack K139 (N16961, A1552, C6706, and HC1037) in chitin estuary microcosms. E7946 was able to compete equally with itself when both strains were lysogens (Fig. 1B; competitive index [CI] = ∼1 for WT versus E7946 CI); however, the ΔK139 nonlysogen was outcompeted when incubated with the E7946 lysogen (Fig. 1B; CI > 1.0 for ΔK139 versus E7946). Furthermore, E7946 was able to outcompete (i.e., CI < 1) all of the clinical isolates that naturally lacked K139 (Fig. 1B). This effect was dependent on K139 viral production, because an E7946 ΔK139 strain was unable to outcompete nonlysogens to the same extent as the WT strain. This is represented by an increase of the competitive index in these cocultures, where a CI of 1 is expected if the two strains compete equally (Fig. 1B). Our results strongly suggest that K139 plays an ecological role by providing a competitive advantage for lysogenic strains in mixed populations containing nonlysogens. By excising within a small fraction of lysogenic cells, and efficiently killing the neighboring nonlysogenic cells via lytic replication, K139 may allow the remaining lysogenic population to successfully compete for resources in a nutrient-limited estuary environment.

10.1128/mSphere.00975-20.1 FIG S1 Growth on insoluble chitin specifically induces K139 lytic replication in an estuarine environment. (A to C) V. cholerae E7946 was grown in chitin microcosms with glucose (0.2%) and lactate (0.5%) added to growth reaction mixtures as indicated. Addition of these readily available carbon sources should diminish the reliance on chitin as a carbon source in these assays. After 24 h of static growth, K139 PFU in culture supernatant (A) and V. cholerae CFU (B) were determined. (C) K139 PFU generated per viable cell plotted as PFU/CFU using the data shown in panels A and B. (D to F) V. cholerae E7946 grown in M9 minimal medium with the sole carbon sources indicated: chitin (insoluble), glucose, lactate, the chitin monosaccharide N-acetylglucosamine (GlcNAc), or soluble β-1,4-linked GlcNAc oligosaccharides. After 24 h of static growth, K139 PFU in culture supernatant (D) and V. cholerae CFU (E) were determined. (F) K139 PFU generated per viable cell plotted as PFU/CFU using the data shown in panels D and E. Through these experiments, we found that the ratio of K139 viral particles per cell was highest during growth on insoluble chitin; thus, V. cholerae may specifically induce K139 production while utilizing insoluble chitin in microcosms. All data are from three independent experiments and plotted as the mean ± SD. Statistical comparisons were made by Student’s two-tailed *t* test by comparing each condition to the control on chitin (*, *P* < 0.05; **, *P* < 0.01; ***, *P* < 0.001; ****, *P* < 0.0001). Download FIG S1, TIF file, 1.1 MB. Copyright © 2020 Molina-Quiroz et al. 2020 Molina-Quiroz et al. This content is distributed under the terms of the Creative Commons Attribution 4.0 International license.

It has previously been shown that some phages can lyse bacterial cells and release intact DNA (8), while other phages degrade host DNA following lysis (9). Release of intact DNA could aid in promoting horizontal gene transfer by NT (8, 10). Here, we have found that K139 promotes neighbor predation under the same chitin microcosm conditions that are required to induce NT. Therefore, we next wanted to test if K139-dependent neighbor predation could promote HGT in a chitin microcosm. To that end, we cocultured a mixture of strains where each carried an unlinked selection marker at a neutral site in the genome (5). The generation of strains with both selection markers indicated HGT. After 48 h of growth in chitin estuary microcosms, the coculture containing E7946 (lysogen, WT) and E7946 ΔK139 (nonlysogen), where neighbor predation is expected to occur, showed the highest number of transformants, which was ∼100-fold higher than a coculture of two K139 lysogens or two nonlysogens where neighbor predation is not expected to occur (Fig. 1C). These results are consistent with neighbor predation promoting HGT.

Prophages have mainly been linked to HGT by transduction (10). However, in chitin microcosms, V. cholerae can also undergo HGT via NT. Thus, next we designed a strategy to determine if the HGT observed is attributable to phage transduction or to NT. To distinguish between these, we inactivated *dprA* in our V. cholerae strains, a gene that is essential for NT but is not required for transduction (11). After 48 h of growth in chitin estuary microcosms, the coculture containing an NT^+^ lysogen (E7946) and an NT^−^ nonlysogen (E7946 ΔK139 Δ*dprA*) showed elevated rates of HGT similar to the coculture containing both NT^+^ strains (Fig. 1C). In contrast, a mixture containing an NT^−^ lysogen and an NT^+^ nonlysogen showed the basal levels of HGT seen in cocultures where no phage predation occurs (Fig. 1C). This suggests that the HGT observed is due to the transfer of DNA from the nonlysogen to the lysogen. This is the opposite of what would be expected for phage transduction where a prophage excised from a lysogenic strain would transduce DNA to the nonlysogen. Together, our results point to an adaptative strategy used by lysogenic strains to induce prophage-dependent neighbor predation in order to thrive and also to capture released DNA for HGT in their aquatic environment.

To the best of our knowledge, our work is the first to establish an ecological role for K139 in enhancing V. cholerae fitness. We show that K139 can enhance survival of V. cholerae lysogens in chitin estuarine environments by neighbor predation of nonlysogens and by driving evolution via HGT. More broadly, our results suggest a novel mechanism by which prophages benefit their lysogenized hosts, which may contribute to the maintenance of these genetic elements in bacterial genomes.

## 

### Bacterial strains and growth conditions.

See Table S1 for a list of all V. cholerae strains used in this study. All strains were routinely grown in Luria-Bertani Miller (LB) broth and agar at 30°C. Where necessary, medium was supplemented with erythromycin (10 μg/ml) or trimethoprim (10 μg/ml).

10.1128/mSphere.00975-20.2 TABLE S1 Strains used in this study. Download Table S1, DOCX file, 0.01 MB. Copyright © 2020 Molina-Quiroz et al. 2020 Molina-Quiroz et al. This content is distributed under the terms of the Creative Commons Attribution 4.0 International license.

For chitin utilization experiments, cells were grown overnight in LB at 30°C with shaking. The next morning, cultures were washed and diluted in 0.7% Instant Ocean (Aquarium Systems) or M9 minimal medium. A 10^5^-CFU amount was inoculated in a 1% shrimp shell chitin (Sigma) suspension in 0.7% Instant Ocean (chitin microcosm) and was incubated up to 48 h at 30°C statically in 14-ml glass test tubes (Fisher Scientific). Lactate or glucose was added to a final concentration of 0.2% and 0.5% when required. GlcNAc sugars were added at 2 mM for pentasaccharides, 3.33 mM for trisaccharides, 5 mM for disaccharides, and 10 mM for monosaccharides. CFU counts were evaluated by serially diluting and plating on LB agar plates.

### Bacteriophage assays.

To assay phage titers, supernatants from bacterial cultures were filtered using 0.2-μm filters (Costar). Filtered supernatants were serially diluted and tittered using E7946 ΔK139 (nonlysogenic, susceptible strain) as described in reference 12. Plates were incubated overnight at 37°C, and turbid plaques were counted the next morning.

### Competition and HGT assays.

Competition assays were conducted in chitin microcosms for 24 h at 30°C statically. Strains were distinguished by *lacZ* phenotype (using *lacZ*^+^ and *ΔlacZ* strain pairs) as previously described (13). Cultures were mixed in a 1:1 ratio and plated for quantitative culture on LB plus X-Gal (5-bromo-4-chloro-3-indolyl-β-d-galactopyranoside). Competitive indices were calculated as previously described (13).

HGT assays between strain pairs were conducted with differentially marked strains where each contained an antibiotic resistance (Ab^r^) marker at a distinct neutral locus (ΔVC1807::Erm^r^ or ΔVCA0692::Tm^r^). As indicated, strains were mixed in a 1:1 ratio in chitin microcosms for 48 h at 30°C statically (5). After 48 h, a portion of each coculture was diluted in LB broth and outgrown for 2 h prior to plating. Reaction mixtures were plated for quantitative culture on trimethoprim (Tm) + erythromycin (Erm) plates to quantify transformants, as well as on Erm and Tm alone to quantify the abundance of each strain within the coculture. Transformation frequency is expressed as CFU of transformants (Erm^r^ + Tm^r^ double resistant)/(CFU of Erm^r^ + CFU of Tm^r^).
