# Supplementary figures and images for: Prophage-Dependent Neighbor Predation Fosters Horizontal Gene Transfer by Natural Transformation
Source: mSphere. 2020 Nov 11;5(6):e00975-20. doi: 10.1128/mSphere.00975-20 (PMC7657591; doi:10.1128/mSphere.00975-20)

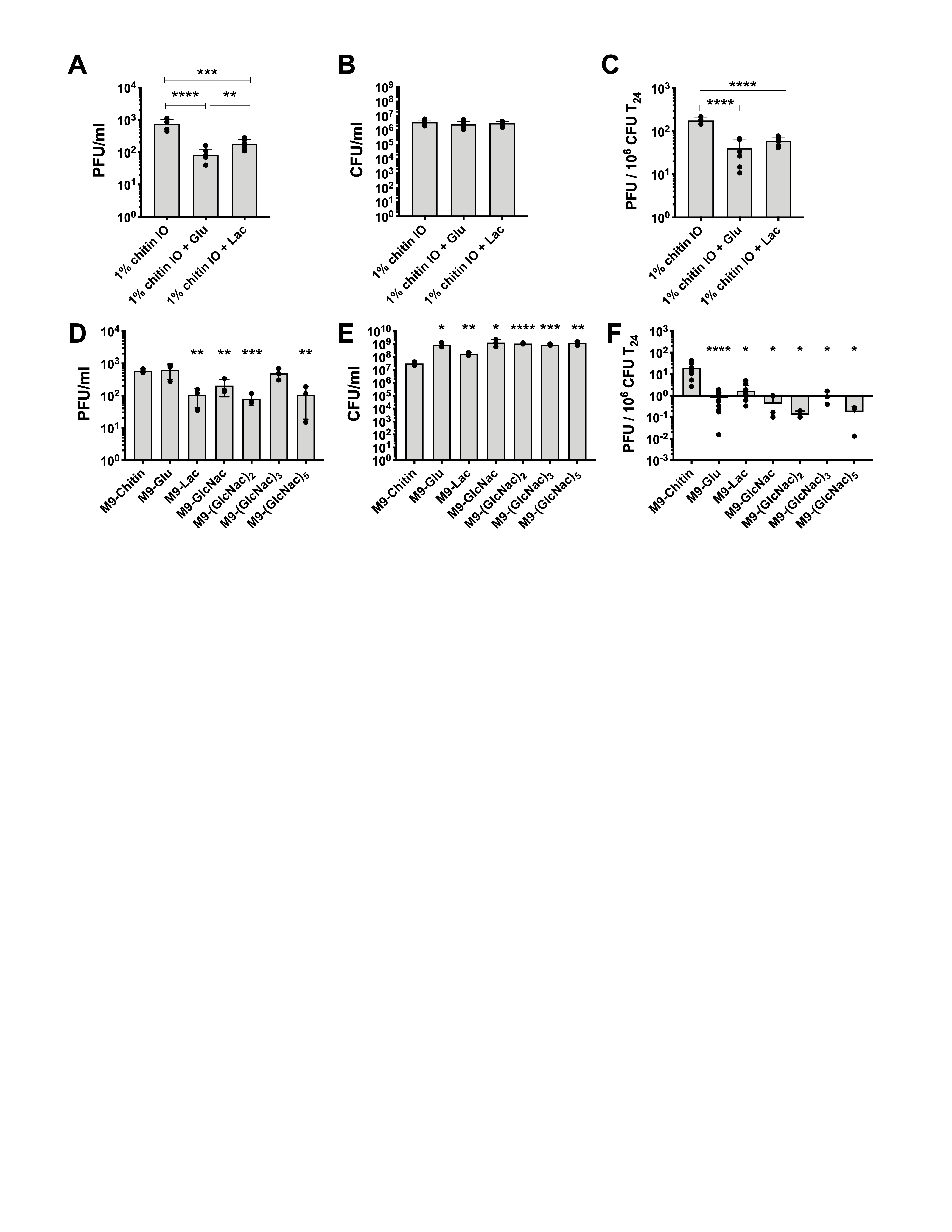

Supplement: FIG S1 [file mSphere.00975-20-sf001.tif]
