# Supplementary material for: Prophage-Dependent Neighbor Predation Fosters Horizontal Gene Transfer by Natural Transformation
Source: mSphere. 2020 Nov 11;5(6):e00975-20. doi: 10.1128/mSphere.00975-20 (PMC7657591; doi:10.1128/mSphere.00975-20)
Supplement: TABLE S1 [file mSphere.00975-20-st001.docx]

| **Name / Strain#** | **Genotype** | **Reference** |
| --- | --- | --- |
| **E7946 WT / AC53** | Spontaneous SmR derivative of E7946, El Tor Biotype | Laboratory collection |
| **A1552** | Wildtype strain | Laboratory collection |
| **C6706** | Wildtype strain | Laboratory collection |
| **HC1037** | Wildtype strain | Laboratory collection |
| **ML111** | E7946 ΔlacZ::KanR | This study |
| **ML137** | E7946 ΔlacZ::KanR, ΔK139::CmR | This study |
| **TND2527** | E7946 ΔVCA0692::TmR, ∆lacZ::SpecR-PrecN-GFP | This study |
| **TND2525** | E7946 ΔVC1807::ErmR, ∆lacZ::SpecR-PrecN-GFP | This study |
| **TND2678** | E7946 ΔVCA0692::TmR, ∆lacZ::SpecR-PrecN-GFP , ΔK139::CmR | This study |
| **TND2543** | E7946 ΔVC1807::ErmR, ∆lacZ::SpecR-PrecN-GFP , ΔK139::CmR | This study |
| **TND2541** | E7946 ΔdprA::ZeoR, ΔVCA0692::TmR, ∆lacZ::SpecR-PrecN-GFP | This study |
| **TND2685** | E7946 ΔdprA::ZeoR, ΔVCA0692::TmR, ∆lacZ::SpecR-PrecN-GFP, ΔK139::CmR | This study |
| **TND2686** | E7946 ΔdprA::ZeoR, ΔVC1807::ErmR, ∆lacZ::SpecR-PrecN-GFP, ΔK139::CmR | This study |
